# Vitamin D binding protein variants associate with asthma susceptibility in the Chinese han population

**DOI:** 10.1186/1471-2350-12-103

**Published:** 2011-08-03

**Authors:** Fei Li, Lei Jiang, Saffron A Willis-Owen, Youming Zhang, Jinming Gao

**Affiliations:** 1 Department of Respiratory Diseases, Peking Union Medical College Hospital, Peking Union Medical College & Chinese Academy of Medical Sciences, Beijing 100730, China; 2 Molecular Genetics and Genomics, National Heart and Lung Institute, Imperial College London, London, UK

## Abstract

**Background:**

Asthma is a genetically heterogeneous disease. Polymorphisms of genes encoding components of the vitamin D pathway have been reported to associate with the risk of asthma. We have previously demonstrated that vitamin D status was associated with lung function in Chinese asthma patients. In this study, we tested whether polymorphisms of genes encoding for vitamin D receptor (*VDR*), vitamin D 25-hydroxylase (*CYP2R1*) and vitamin D binding protein (*GC*) were associated with asthma in the Chinese Han population.

**Methods:**

We sequenced all 8 exons of *VDR *and all 5 exons of *CYP2R1 *in a Chinese case-control cohort of asthma consisting of 467 cases and 288 unrelated healthy controls. Two mutations were identified in these regions. These variants were specified as rs2228570 in exon 2 of *VDR *and rs12794714 in exon 1 of *CYP2R1*. We also genotyped two common polymorphisms in *GC *gene (rs4588 and rs7041) by a PCR-restriction fragment length polymorphism (RFLP) method. We analyzed the association between these 4 polymorphisms and asthma susceptibility and asthma-related traits.

**Results:**

Polymorphic markers in *VDR *and *CYP2R1 *were not associated with asthma in the Chinese Han cohort. Importantly, variants in *GC *gene, which give rise to the two most common electrophoretic isoforms of the vitamin D binding protein, were associated with asthma susceptibility. Compared with isoform Gc1, Gc2 was significantly associated with the risk of asthma (OR = 1.35, 95% CI = 1.01-1.78 p = 0.006).

**Conclusions:**

The results provide supporting evidence for association between *GC *variants and asthma susceptibility in the Chinese Han population.

## Background

Asthma is a chronic inflammatory disease of the airways characterized by reversible airflow obstruction and airway hyper-reactivity. The disease exhibits complex patterns of transmission, and has an estimated heritability of 36 - 79% [1]. To date, a plethora of genes and variants have been implicated in the pathogenesis and progression of asthma [2,3]. However, relatively few of these genes and variants have been successfully replicated.

In recent years the vitamin D pathway has emerged as a new potential component of asthma and allergy susceptibility. The majority of this evidence has been derived from epidemiological surveys. Deficiency of vitamin D has been found to increase the risk of asthma or asthma exacerbation [4-8]; higher maternal vitamin D intake during pregnancy has been associated with a reduced risk of wheezing illnesses in young children [9-11]; and circulating 25-hydroxyvitamin D (25OHD) has been associated with lung function in patients with asthma [7,12,13]. More recently, a small randomized double-blinded 6 month trial indicated that vitamin D supplementation in children may reduce asthma exacerbation following acute respiratory infection [14].

At the genetic level, a number of polymorphisms have been identified in various components of the vitamin D pathway; including the vitamin D receptor *VDR*, the microsomal vitamin D hydroxylase *CYP2R1*, and the vitamin D binding protein *GC*. *VDR *is the major receptor for the bioactive form of 25OHD, 1α,25-dihydroxyvitamin D3 (1,25OHD). The gene encoding *VDR *is located on human chromosome 12q13 and has 8 coding exons encode 427 amino acids. *CYP2R1*, positioned on human chromosome 11p15, is responsible for transforming photo-synthesized and dietary vitamin D into 25OHD [15]. *CYP2R1 *gene has 5 exons encoding 501 amino acids. Vitamin D metabolites in the circulation are bound to vitamin D binding protein (DBP) encoded by a highly polymorphic gene *GC*. *GC *is located on chromosome 4q13 and has 13 exons encoding 474 amino acids. There are two common functional single nucleotide polymorphisms (SNPs) in exon 11 of *GC*, which are rs4588 and rs7041 positioned at codons 416 and 420. These variants have been associated with different binding affinity for 25OHD, and give rise to the two most common electrophoretic isoforms of DBP; Gc1 (which can be further subdivided into Gc1F and Gc1S); and Gc2. Whilst *GC *isoforms have been implicated in chronic obstructive pulmonary disease (COPD) across a variety of contrasting population (for review, see reference 16), there is relatively little evidence of their involvement in asthma [16,17]. *VDR *variants have been extensively studied in asthma, although the results have shown contradictory [15,18-23]. Similarly, evidence for association between *CYP2R1 *variants and asthma has also been inconsistent between populations [15]. One potential explanation for this inconsistency may be differences in the underlying pattern of linkage disequilibrium between typed markers and the true disease loci in distinct populations.

We previously showed that lower vitamin D status in serum was associated with impaired lung function in Chinese asthma patients [12]. In this study we set out to evaluate whether the variants of genes encoding for the key components of vitamin D pathway, including *CYP2R1*, *VDR*, and DBP, were associated with asthma or asthma-related phenotypes in a case-control design study in the Chinese Han population.

## Methods

### Subjects and phenotypical characteristics

We recruited 467 asthma patients and 288 controls with Han ethnicity from the Northern region of China. All participants were unrelated. Asthma cases were recruited at the Pulmonary Clinic, Peking Union Medical College Hospital. Ethnicity-matched controls were selected from non-asthmatic, non-atopic, healthy individuals with normal lung function [24].

Measurement of serum total IgE was performed by applying the UniCAP System (Pharmacia, Uppsala, Sweden) according to the manufacturer's instructions.

Asthma was diagnosed using the criteria defined by international guideline [25]. All cases complained of current asthma symptoms including wheezing, cough, awakening at night and shortness of breath. Asthmatics with forced expiratory volume in 1s (FEV_1_) ≥ l70% of predicted underwent the methacholine challenge test, and positive bronchial hyperresponsiveness was defined by a 20% fall in FEV_1 _at inhaled methacholine concentrations ≤16 mg/ml. Patients with FEV_1 _< 70% of predicted underwent an airway reversibility test with positive airway reversibility defined by both 12% improvement in FEV_1 _and an increase ≥ 200 ml in the absolute FEV_1 _value after β_2_-agonist inhalation [25].

The incidence of asthma was higher in females than in males (60.4% vs 39.6%). The baseline FEV_1_% predicted was significantly lower in asthmatics than normal controls (68.9 ± 37.9% vs 90.6 ± 14.9%, *P *< 0.001). Methacholine airway challenge test was performed in 224 (48%) asthma subjects with baseline FEV_1_> 70% of predicted; in all of these subjects, positive bronchial hyperresponsiveness was documented. The airway reversibility test was carried out in 273 patients (58%). The mean improvement in FEV_1 _was 26.4 ± 14.3%. The serum total IgE values in controls were within the normal range. Detailed characteristics of the individuals are listed in Table 1.

**Table 1:** Demographics of the participants

	Asthma (n = 467)	Control (n = 288)
Mean age, years	40.8 ± 14.1	45.1 ± 0.7
Range	18-76	22-72
Sex, n (%)		
Male	185 (39.6)	158 (55.0)
Female	282 (60.4)	130 (45.0)
log_e_IgE, U/L	5.24 ± 1.44	3.64 ± 1.10
Eosinophils,%	1.63 ± 0.91	2.19 ± 0.93
Lung function		
FEV_1_,%	68.94 ± 37.88	90.6 ± 14.92
FEV_1_/FVC,%	63.93 ± 14.91	81.53 ± 9.52

The study protocol was reviewed and approved by the Human Research Ethics Committee of the Peking Union Medical College Hospital, and all subjects gave written informed consent to participate in the study.

### SNPs selection and genotyping

DNA samples (derived from 467 asthma patients and 288 healthy, non-atopic controls) were extracted from peripheral blood leukocytes using a standard phenol-chloroform method.

All selected genes code for key enzymes or components responsible for vitamin D metabolic pathway. Single nucleotide polymorphisms (SNPs) of genes tested in this study have been associated with asthma susceptibility [15]. We directly sequenced the exons of *VDR *and *CYP2R1 *in all 755 individuals and identified two mutations, specified as rs2228570 in exon 2 of *VDR *and rs12794714 in exon 1 of *CYP2R1*. Sequencing *VDR *and *CYP2R1 *genes was performed at the Beijing Genomics Institute (http://www.genomics.cn). DNAStar 5.0 SeqMan Software was used to identify DNA variants and provide genotype calls. The primers for PCR were designed by using Primer Premier 5.0 Software. Sequences of the primers are listed in Table 2.

**Table 2:** Primer sequences and PCR conditions for genotyping *VDR, CYP2R1 *and *GC *variants

Gene	Region	PCR primers(5'→3')	Sequencing primer(5'→3')	Annealing temperature(°C)	PCR product (bp)	Restriction Enzyme	RFLP products (bp)
*VDR*							
	Exon 1	F:TGGCACCAAGGATGCCAGCT		57	364		
		R:TCCTGCTCCTGTGGCTGTGA	R:GTGAGCGCCGCATGTTCC				
	Exon 2	F:CCTCATGTCTTCTGTTGGAG	F:CCTCATGTCTTCTGTTGGAG	57	438		
		R:TGCATCTGACCCTGGACTTC					
	Exons 3,4	F: CCTATCTTGGACCTTTACCC	F: CCTATCTTGGACCTTTACCC	57	758		
		R:TCCATTAGGGAGCCTTCCAC					
	Exon 5	F: CTGTGGAGTCACTGTGGGAT	F: CTGTGGAGTCACTGTGGGAT	57	390		
		R:GAAGTGGTGGATGAGTGATC					
	Exons 6,7	F: TGTTGGTGCCCAGCAGGTGT	F: TGTTGGTGCCCAGCAGGTGT	57	680		
		R:GCTACGTCTCCCTTCAGGTT					
	Exon 8	F: TGCTGCCGTTGAGTGTCTGT		57	526		
		R:GGTTGGACAGGAGAGAGAAT	R:ACAGGAGAGAGAATGGGCTG				
*CYP2R1*							
	Exon 1	F:GGGACCTGAGGGTATCCTGC	F:ACCTGAGGGTATCCTGCCAATG	55	409		
		R:GGCGGCCATAAGTCCAACC					
	Exon 2	F: AGGGACGTAACAAATCCTGG	F: AGAGCCGCACTTACCACTTG	57	412		
		R: GTAGTACAGCCTGAAAGGTC					
	Exon 3	F: AACAGGACCCAACCATGTAG	F: GGAGGACAATTTGGAGAAGG	57	881		
		R: CATCGCAGGAGTTCCTAAAG					
	Exon 4	F: TGGCTTGTTATAGGTTATCC		57	574		
		R: CATTCTCTCCTGTTAGAATC	R: CATTCTCTCCTGTTAGAATC				
	Exon 5	F: GTTCTGCTTGCTGAAGTGTC	F: CATAGTTCCCTCTTTCTTTG	57	415		
		R: ACCAAGTTCAGGGATAAGGC					
*GC*							
	rs4588 rs7041	F:AAATAATGAGCAAATGAAAGAAGAC		55	483	Hae III	297/186
		R:CAATAACAGCAAAGAAATGAGTAGA				Sty I	305/178

We genotyped the two most commonly studied SNPs in the *GC *gene, rs4588 and rs7041, using a standard PCR-restriction fragment length polymorphism (PCR-RFLP). Genetic variants in *GC *gene have been shown the different affinity in binding vitamin D metabolites [26]. The SNPs rs4588 and rs7041 have been found to have associations with several chronic lung diseases, such as COPD and tuberculosis [27,28]. Carriage of the genotype of Gc2 allele was associated with increased tuberculin-stimulated IFN-γ release in Gujarati Asian TB contacts [27]. Homozygous carriers of the rs7041 T allele exhibited an increased risk for COPD in a Belgium population [28]. There are only 11 base pairs apart between these two SNPs in the human genome, however, they are not in the same LD block. Thus we selected these two important SNPs in our Chinese samples to test the association with the risk of developing asthma. These two SNPs, rs4588 and rs7041, are positioned at codons 416 (GAT→GAG, Asp→Glu) and 420 (ACG→AAG, Thr→Lys) of exon 11 of the *GC *gene (Table 3) [27]. The different loci were recognized by the following restriction endonucleases (New England Biolabs): Hae III for T/G at 37°C, Sty I for C/A at 37°C.

**Table 3:** Deduction of vitamin D binding protein genotypes from Haelll and Styl genotypes [27]

Haelll/Styl Genotypes	Potential haplotypes	Deduced haplotypes	Corresponding DBP genotypes	Asthma (N)	Control (N)
HH SS	HS/HS	HS/HS	Gc1F/Gc1F	72	41
HH Ss	HS/Hs	HS/Hs	Gc1F/Gc2	129	59
HH ss	Hs/Hs	Hs/Hs	Gc2/Gc2	24	1
Hh SS	HS/hS	HS/hS	Gc1F/Gc1S	93	46
Hh Ss	Hs/hS or HS/hs*	Hs/hS	Gc2/Gc1S	71	35
hh/SS	hS/hS	hS/hS	Gc1S/Gc1S	27	18
Hh/Ss	hS/hs*	Gc1s/not deduced	Not assigned	0	0

*frequency of the hs haplotype is very low due to linkage disequlibrum between loci

The PCR reactions were performed in a total volume of 30 μl (40 ul for *GC *variants genotyping) containing 70 ng of genomic DNA, 3 μl of 10 × PCR Buffer, 1.5 μM of MgCl_2_, 0.17 mM of dNTPs, 100 pM of each primer, and 1.5 U of Taq DNA polymerase (Takara). In general, PCR cycle condition consisted of an initial denaturation step at 94°C for 3 min followed by 30 cycles (35 cycles for GC variants genotyping) of denaturation for 30 s at 94°C, annealing for 30 s at 55-57°C, extension for 40- 45 s at 72°C, and a final extension at 72°C for 7 min.

### Statistical analysis

Because of similar functional characteristics of *GC *isoforms Gc1F and Gc1S, allele carriers were combined to produce a total of 3 genotypes: Gc1/1, Gc1/2 and Gc2/2. Allele and genotype frequencies of polymorphisms in *VDR*, *CYP2R1 *and *GC *were obtained by direct counting, and Hardy-Weinberg equilibrium was evaluated by a chi-squared test (using SPSS statistical software, version 12.0). All genotype and phenotype data were formatted using PLINK.

Quantitative traits were tested using a variance components approach implemented in the statistical package Merlin [29]. Quantile normalization was applied to all quantitative traits prior to analysis (with the exception of total serum IgE which had already been subjected to a log transformation). Missing genotypes were inferred using the --- infer option available in Merlin.

In addition, an unpaired t-test was used to compare the baseline data between asthma patients and healthy controls. One-way ANONA was used to assess the genotype distribution in asthma patients. A Bonferroni correction was used to adjust p-values for multiple testing (based on an alpha threshold of 0.05). Genotype and allele frequencies in cases and controls were also compared by contingency table analysis. Genotype relative risk was calculated according to the statistical method described by Lathrop [30]. This method compares case genotype frequencies with expected control genotype frequencies under the assumption of Hardy-Weinberg equilibrium, and is more powerful than standard contingency table analysis.

## Results

We genotyped 1 SNP (rs12794714) in *CYP2R1*, 1 SNP (rs2228570) in *VDR*, and 2 SNPs (rs4588 and rs7041) in *GC*. The allele and genotype frequencies are listed in Table 4. All SNPs met criteria for Hardy-Weinberg equilibrium in both case and control groups (all p-value > 0.05).

**Table 4:** Association between asthma and polymorphisms of *CYP2R1*, *VDR*, *GC *genes

	Genotype/Allele	**Asthmatics**, n (%)	Controls, n (%)	χ^2^	*P*
*VDR *rs2228570					
	CC	152	60		
	CT	230	91		
	TT	85	37	0.194	0.908
	C	534 (57.2)	211 (56.1)		
	T	400 (42.8)	165 (43.9)	0.122	0.727
*CYP2R1 *rs12794717					
	CC	171	64		
	CT	214	86		
	TT	81	25	1.02	0.601
	C	556 (59.7)	214 (61.1)		
	T	376 (40.3)	136 (38.9)	0.234	0.628
*GC*					
	Gc1/1	192	105		
	Gc1/2	200	94		
	Gc2/2	24	1	10.401	**0.006**
	Gc1	584 (70.2)	304 (76.0)		
	Gc2	248 (29.8)	96 (24.0)	4.527	**0.033**

No significant difference was observed in the genotype and allele frequencies of rs2228570 in *VDR *and rs12794714 in *CYP2R1 *between asthmatic cases and controls (Table 4), suggesting that these SNPs were not genetically associated with the risk of asthma.

Importantly, single marker analysis of association showed that *GC *polymorphism (expressed as Gc1/1, Gc1/2 and Gc2/2) was significantly associated with asthma susceptibility in our Chinese samples, the risk conferred by it was slightly elevated (OR = 1.35, 95% CI = 1.01 - 1.78, P = 0.006). Compared with Gc1/1 genotype, Gc2/2 genotype was strongly associated with the risk of asthma (OR = 13.13, 95% CI = 2.42-7.13, P = 0.001) (Table 4). These results may suggest that Gc2/2 genotype confers a significant risk for developing asthma.

Vitamin D deficiency has been defined as serum 25OHD < 50 nmol/l [31]. We have previously demonstrated that vitamin D deficiency is common among Chinese patients with asthma [12]. Thus, we retrospectively analysed the association between the *GC *polymorphisms and serum 25-OHD concentrations, however, no significant difference was observed (Table 5).

**Table 5:** Frequency of genotypes of *CYP2R1, VDR, DBP *in asthmatics with different vitamin D status

Serum 25OHD	Subjects, n	Genotypes, n (%)			*P *value
		***CYP2R1 *rs12794717**			
		**CC**	**CT**	**TT**	

≥50 nmo l/L	49	20 (40.8)	24 (49)	5 (10.2)	
< 50 nmol/L	363	132(36.4)	167 (46)	64 (17.6)	0.42
					
		*VDR *rs2228570			
		CC	CT	TT	

≥50 nmol/L	49	13 (26.5)	25 (51.1)	11 (22.4)	
< 50 nmol/L	362	124 (34.3)	180 (49.7)	58 (16)	0.397
					
		*GC *rs4588 and rs7041			
		Gc1/1	Gc1/2	Gc2/2	

≥50 nmol/L	49	20 (40.8)	26 (53.1)	3 (6.1)	
< 50 nmol/L	363	169 (46.5)	173 (47.7)	21 (5.8)	0.747

We further examined the evidence for association between the polymorphic markers in these three genes and a number of quantitative asthma-related traits using Merlin. We were not able to demonstrate any significant associations between the studied SNPs and blood eosinphil counts, log-transformed serum IgE or lung function measures, even after stratification by sex (data not shown).

## Discussion

We performed a case-control study assessing association between polymorphisms in 3 genes of the vitamin D pathway and asthma susceptibility in the Chinese Han population. We showed that the *GC *polymorphisms giving rise to the major electrophoretic isoforms of vitamin E binding protein (rs4588 and rs7041) were significantly associated with the risk of asthma, although not 25OHD concentrations in asthma patients. By sequencing all exons of *VDR *and *CYP2R1 *genes, we have identified that the full range of exonic genetic variants presented in our cohort have shown allele frequencies ≥0.13%. We observed only two exonic SNPs, rs2228570 (also known as Fok I) in *VDR *and rs12794714 in *CYP2R1*, both of which had been previously characterised. No association could be identified between either of these SNPs and asthma. Because exonic regions are more likely to carry variants that disrupt protein function [32,33], our polymorphism screening did not include intronic or promoter regions of these genes which may also potentially contain causal variants [34].

The FokI polymorphism in exon 2 of *VDR *consists of a T to C change which has been reported to cause gene-specific and cell type-specific effects. *VDR *polymorphisms have been widely studied in asthma, and the results have been contradictory [15,18-23]. The association of polymorphisms in *VDR *with asthma was first reported in Northern American family-based studies [19,20], but initial positive results were not replicated in subsequent populations [22,23]. Consistent with our negative results, a previous case-control study of rs2228570 performed in a Chinese Han population also failed to show an association with asthma [21].

The *CYP2R1 *variant, rs12794714, has previously been associated with serum 25OHD levels in a healthy Caucasian population [35]. Alternative *CYP2R1 *variants have also been implicated in asthma susceptibility, though these effects have not replicated consistently among populations [15]. This lack of consistency may reflect a linkage disequilibrium phenomenon (with non-coding regulatory variants(s)), a consequence of complex, undefined gene-gene (epistatic) or gene-environment interactions, or a low magnitude effect requiring large, highly powered cohorts for its detection.

The vitamin D binding protein (DBP), encoded by *GC*, is the principle plasma carrier protein of vitamin D and its metabolites. Due to a higher binding affinity of Gc1F and Gc1S to 25OHD, the isoforms are hypothesized to deliver 25OHD to target tissues more efficiently than Gc2 [26]. The results presented here indicate that the two common SNPs in *GC *which determine DBP isoforms associate significantly with susceptibility to asthma, and that the Gc1 allele might confer a protective effect. One previous study of *GC *variants in asthma did not find an association, however, this study included a very small number of participants and was probably underpowered to detect a difference [17].

Polymorphisms in *GC *gene have been found to be significantly associated with 25OHD concentrations by genome-wide association (GWA) [36,37]. We have previously shown that lower serum 25OHD concentrations are associated with impaired lung function measurements in Chinese patients with asthma [12]. In this study, we reported the association between genetic variants in *GC *and asthma susceptibility. Thus, we further investigated the influence of *GC *genotypes on serum 25-OHD concentrations in asthma patients. When performing multiple tests for the association analysis with Bonferroni correction to the dataset, we couldn't find any significant association. One explanation could be that circulating serum 25OHD concentrations are influenced by several genetic loci [36,37].

## Conclusion

In conclusion, this study has shown that genetic variants in the gene encoding the vitamin D binding protein (*GC) *contribute to asthma susceptibility in a Han Chinese population. The OR in this study with respect to Gc2/2 homozygote suggests a modest but definite genetic effect. We have not found associations between that exonic variants in the *VDR *and *CYP2R1*genes and asthma or asthma-related traits in our Chinese population. Further research is needed to understand how gene-gene or gene-environment interactions and causative regulatory variants (intronic, promoter, or trans-acting) influence vitamin D metabolism in asthma patients [34].

## Competing interests

The authors declare that they have no competing interests.

## Authors' contributions

FL performed the whole procedure of the experiments. LJ helped in performing the experiment. SAWO and YZh performed the statistical analysis and drafted the manuscript. JG designed and supervised the experiment, and drafted the manuscript. all authors read and approved the final manuscript.

## Pre-publication history

The pre-publication history for this paper can be accessed here:

http://www.biomedcentral.com/1471-2350/12/103/prepub
